# Genome-Scale Metabolic Model of *Actinosynnema pretiosum* ATCC 31280 and Its Application for Ansamitocin P-3 Production Improvement

**DOI:** 10.3390/genes9070364

**Published:** 2018-07-20

**Authors:** Jian Li, Renliang Sun, Xinjuan Ning, Xinran Wang, Zhuo Wang

**Affiliations:** 1 Bio-X Institutes, Key laboratory for the Genetics of Developmental and Neuropsychiatric Disorders (Ministry of Education), Shanghai Jiao Tong University, Shanghai 200030, China; lijian1992@sjtu.edu.cn (J.L.); sunrenliang@sjtu.edu.cn (R.S.); 2 School of Life Sciences and Biotechnology, Shanghai Jiao Tong University, Shanghai 200040, China; qidie@sjtu.edu.cn (X.N.); shirley.wxr@sjtu.edu.cn (X.W.); 3 State Key Laboratory of Microbial Metabolism, Shanghai Jiao Tong University, Shanghai 200040, China

**Keywords:** *Actinosynnema pretiosum* ATCC 31280, genome-scale metabolic model, ansamitocin P-3, methionine metabolism, metabolic shift

## Abstract

*Actinosynnema pretiosum* ATCC 31280 is the producer of antitumor agent ansamitocin P-3 (AP-3). Understanding of the AP-3 biosynthetic pathway and the whole metabolic network in *A. pretiosum* is important for the improvement of AP-3 titer. In this study, we reconstructed the first complete Genome-Scale Metabolic Model (GSMM) *Aspm1282* for *A. pretiosum* ATCC 31280 based on the newly sequenced genome, with 87% reactions having definite functional annotation. The model has been validated by effectively predicting growth and the key genes for AP-3 biosynthesis. Then we built condition-specific models for an AP-3 high-yield mutant NXJ-24 by integrating *Aspm1282* model with time-course transcriptome data. The changes of flux distribution reflect the metabolic shift from growth-related pathway to secondary metabolism pathway since the second day of cultivation. The AP-3 and methionine metabolisms were both enriched in active flux for the last two days, which uncovered the relationships among cell growth, activation of methionine metabolism, and the biosynthesis of AP-3. Furthermore, we identified four combinatorial gene modifications for overproducing AP-3 by in silico strain design, which improved the theoretical flux of AP-3 biosynthesis from 0.201 to 0.372 mmol/gDW/h. Upregulation of methionine metabolic pathway is a potential strategy to improve the production of AP-3.

## 1. Introduction

**Figure 1:**
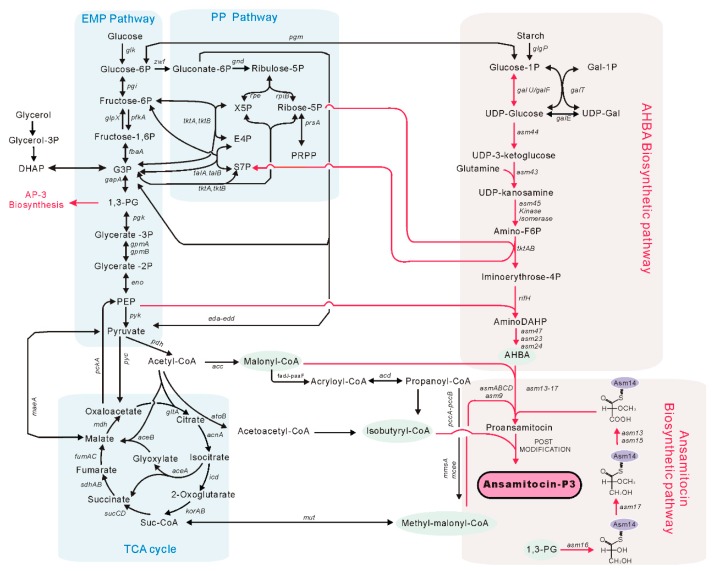
Biosynthetic pathway of ansamitocin P-3 in *Actinosynnema pretiosum*. AP-3: ansamitocin P-3; EMP pathway: Embden Meyerhof Parnas pathway; PP pathway: pentose phosphate pathway; G3P: glyceraldehyde 3-phosphate; PEP: phosphoenolpyruvate; DHAP: dihydroxyacetone phosphate; X5P: D-xylulose 5-phosphate; UDP: uridine diphosphate; Gal: alpha-d-Galactose; E4P: erythrose 4-phosphate; S7P: sedoheptulose 7-phosphate; PRPP: 5-phosphoribosyl 1-pyrophosphate; AHBA: 3-amino-5-hydroxybenzoic acid; F6P: fructose-6-phosphate; TCA cycle: tricarboxylic acid cycle.

*Actinosynnema pretiosum* ATCC 31280 was isolated in 1977 [1] and is known as the producer of ansamitocins [2]. Ansamitocins are a series of complex polyketide compounds [3], among which ansamitocin P-3 (AP-3) was confirmed to be the most potent antitumor agent [4,5]. Recently, AP-3 has been used as the payload in many antibody-drug conjugants, such as trastuzumab emtansine, which was approved by the FDA for breast cancer treatment [6]. Although the antitumor activity of AP-3 is highly effective, the commercial application of AP-3 is substantially limited by its low production titer [7]. Therefore, in the past decades, many efforts have been made to improve the production of AP-3 [8,9,10]. These strategies includes mutant screening, medium optimization, and genetic engineering. However, the titer of AP-3 is still far from ideal. The reason for limited success in the improvement of AP-3 titer is probably due to a less understanding of the AP-3 biosynthetic pathway, which involves multiple metabolic pathways (Figure 1) and the whole metabolic network in *A. pretiosum* [11].

Genome-Scale Metabolic Models (GSMMs) play important roles in systems biology [12,13], which aim at understanding complex interactions between genes, proteins, and metabolites by integrating and modeling multiple data sources [14]. GSMMs can predict the theoretical maximum production of a particular compound and simulate the effects of genetic modification on growth and target compound production, which provides links between genotype and phenotype of an organism [15,16]. GSMMs have been widely used in studies of overproducing target products. For example, using *Esterichia coli* GSMM *i*JR904 [17], Choi et al. found that microaerobic condition was more efficient than aerobic condition in achieving higher titer of 4-hybroxybutyric acid in *E. coli*. The yield of 4-hybroxybutyric acid was increased from 35.2 g/L to 103.4 g/L [18]. Kuhn Ip et al. used GSMM *i*JO1366 [19] to explore the genetic manipulation strategies for overproducing fatty acids in *E. coli*, resulting in a 5.4-fold increase by the knockout of gene *fadE* [20]. Brochado A.R. et al. achieved a five-fold increase in vanillin production in baker’s yeast by the overexpression of *O*-methyltransferase based on genome-scale modeling approach [21]. Wu et al. increased acarbose production by 78% in *Actinoplanes* ssp. SE50/110 based on the predicted genetic modification strategies [22].

In this study, we reconstructed and validated the first GSMM of *A. pretiosum* ATCC 31280 based on the newly sequenced genome (Genebank accession number: CP029607). Then we integrated the model with time-course transcriptome data of a high-yield mutant strain NXJ-24 [23] to investigate the change of metabolic flux distribution during the fermentation process. Furthermore, potential strategies for improving AP-3 production were predicted by in silico strain design based on the established model.

## 2. Materials and Methods

### 2.1. Reconstruction of the Genome-Scale Model of Actinosynnema pretiosum ATCC 31280

The genome-scale metabolic model of *A. pretiosum* ATCC 31280 was reconstructed based on the newly sequenced genome, by a complicated process of annotation, transformation, gap filling, and refinement. Genome annotation was performed through RAST server [24], and then the draft model was reconstructed by ModelSEED [25] with the annotation. Public databases, such as KEGG, were used to manually refine the draft model, including addition of specific reactions, such as biosynthetic reactions for the biomass and AP-3, modification of the reversibility of core metabolic reactions, deletion of incorrect reactions, and filling of metabolic gaps. Some reactions obtained from published literatures were also incorporated into the model. The final reconstructed GSMM of *A. pretiosum* ATCC 31280 has 1282 genes, we called the model *Aspm1282*.

### 2.2. Biomass Composition in Aspm1282 Model

Knowledge of the cellular biomass composition is an important prerequisite for the in silico flux analysis, especially during the exponential growth phase, where the primary cellular objective is to maximize growth [26]. There is no detailed information about the biomass composition for *A. pretiosum*, we obtained each component and corresponding proportion through measurement and literature mining. The cellular compositions of *A. pretiosum*, consisting of lipids, proteins, carbohydrates and so on, were mostly obtained from text books [27]. The dry cell weight (DCW), consumption rate of sugar, and the yield of AP-3 were determined by our experiment. The DNA composition was calculated by using the G+C content. The RNA composition was calculated based on the presumptive percentage of messenger RNA (mRNA), ribosomal RNA (rRNA), and transfer RNA (tRNA) was 5%, 75%, and 20%, respectively. The compositions of biomass for *A. pretiosum* ATCC 31280 are listed in Table S2 (Supplementary files).

### 2.3. Flux Balance Analysis

To perform in silico simulations and predict the metabolic characteristics of *A. pretiosum*, constraint-based flux analysis was carried out under the assumption of a pseudo-steady state [28]. For growth simulation, we set the biomass equation as the objective function to predict the growth rate and flux distribution, the optimization problem is formulated as following:

(1) $$\underset{v}{\mathrm{maximum}:\;}biomass\;\mathrm{subject}\;\mathrm{to}:\;S\cdot v=0\;a_{j}\;\Delta \leq v\leq b_{j}$$

v is a flux vector representing a particular flux configuration, S is the stoichiometric matrix, a*_j_* and b*_j_* are the minimum and maximum fluxes through reaction *j*. We also investigated the metabolic capacity of *A. pretiosum* in AP-3 production by setting AP-3 flux as the objective function. The flux balance analysis (FBA) simulation was performed using COBRA Toolbox [29] with Gurobi [30] as the linear programming solver.

### 2.4. Actinosynnema pretiosum NXJ-24 Mutant and the RNA-Seq Data across Fermentation Process

In our previous study, the NXJ-24 mutant was generated by knockout *ansa30* gene, and overexpressing *asm10* gene [10]. In the disruption of *ansa30* gene, two 1.5-kb homologous arms for *ansa30* disruption were respectively amplified with primers Del30-L-F/R and Del30-R-F/R, sequenced, and together cloned to SpeI/EcoRI-digested plasmid pJTU1278 to give the *ansa30*-inactivation plasmid pLQ578. In the overexpression of *asm10*, the kasOp promoter was cloned into BamHI/SpeI-digested plasmid pDR3 [31] to generate plasmid pDR3-K. Genes of *asm10* were amplified with primers *asm10*-F/R. Subsequently, the sequenced genes were individually inserted into SpeI/EcoRI-digested plasmid pDR3-K under the control of kasOp promoter, generating plasmids pLQ586 and pLQ589. The recombinant plasmids were introduced into NXJ-24 from *E. coli* ET12567 (pUZ8002) through intergeneric conjugation. The transcriptome data of NXJ-24 during fermentation process of day 1, day 2, day 3, and day 5 were sequenced by Shanghai Biotechnology Corporation, Shanghai, China. We also sequenced the transcriptome of the wild-type strain in day 5, which was used to construct decline-phase specific model of the wild-type for strain optimization.

### 2.5. Pathway Enrichment Analysis for Essential Genes and Active Reactions in Aspm1282 Model

We used enrichment analysis to identify pathways enriched in essential genes for AP-3 biosynthesis and active reactions during fermentation process significantly. The hypergeometric test was used to calculate the *p*-value of each pathway (Equation (2)).

(2) $$p_{j}=1-\sum_{i=0}^{m-1}\frac{\left(\begin{array}{c} M_{j}\\ i \end{array}\right)\left(\begin{array}{c} N-M_{j}\\ n-i \end{array}\right)}{\left(\begin{array}{c} N\\ n \end{array}\right)}$$

*p_j_* is the *p*-value of subsystem *j*, *N* is the total number of reactions/genes in *Aspm1282* model, *M_j_* is the number of reactions/genes in subsystem *j*, *n* is the number of essential genes or active reactions in the whole model, *m* is the number of essential genes or active reactions in subsystem *j*.

### 2.6. E-FLUX Method for Condition-Specific Model Construction by Integrating Gene Expression Data

E-FLUX is a method for modeling metabolic states under specific conditions by integrating gene or protein expression data, which extends the technique of FBA by modeling maximum flux constraint as a function of measured gene expression [32]. Here we set the upper bound b*_j_* of each reaction as the expression value of the genes encoding enzymes catalyzing reaction *j*.

(3) $$exp_{i}=\log\left({\mathrm{FPKM}}_{i}+1\right)$$

(4) $$b_{j}=\sum_{i\in {\mathrm{rxn}}_{j}}exp_{i}$$

FPKM*_i_* is the FPKM (fragments per kilobaseof exon per million fragments mapped) of gene *i* measured by experiment, *exp_i_* represents the expression level of gene *i*, b*_j_* represents the upper bound of reaction *j*, rxn*_j_* represents the reaction *j*.

By this conversion, we set constraints on both lowly and highly expressed reactions, then FBA will be run by fulfilling the same objective function.

### 2.7. In Silico Strain Design Approach OptRAM

OptRAM (optimization of regulatory and metabolic network) is a meta-heuristic strain optimization method based on GSMM, which can be divided into three parts: prediction, random modification, simulation, and evaluation. Firstly, we use parsimonious enzyme usage FBA (pFBA) [33] to predict the flux value of each reaction. To identify the global optimal genetic modification strategy, we used simulated annealing [34,35], which is able to accept a worse solution in the early stage (avoiding getting stuck in local maximal). In each iteration of simulated annealing:

1. Randomly pick up one gene and assign a random mutation code to it. As shown in Table 1, code represents the manipulation of the mutation on selected gene, and FC is the corresponding fold change of the mutated gene expression, which reasonably simulates the up-regulation, down-regulation, or knockout of particular gene.
2. The expression of genes will be transformed to corresponding reactions in the metabolic model through changing the upper bound or lower bound of it. The range of changes is based on the reference flux values from pFBA.
3. The phenotype of modified strain was simulated using FBA, and the objective value was used to evaluate the mutant. OptRAM will choose to go back to last round of iteration or accept this solution according to the Metropolis criterion [35]:
  (5) $$P=e^{\frac{\Delta f}{T\left(K\right)}}$$
  *T* (k + 1) = *T* (k) × α (6)
  where Δf is the difference between objective score of new solution and the previous one, *T* is the control parameter, α is the attenuation factor (α < 1). When Δf > 0 or P > R, new objective score is accepted as the new solution, otherwise OptRAM will go back to the original solution. When OptRAM gets a new solution, *T* (k + 1) = *T* (k) × α will be carried out. When a certain number of iterations is reached, the solution at that moment will be the final strain genetic modification solution.

## 3. Results

### 3.1. Reconstructed Genome-Scale Metabolic Model of A. pretiosum

The composition of model *Aspm1282* is shown in Table 2, including 1282 genes, 1614 metabolites, and 1669 reactions (1520 intracellular metabolic reactions, 148 transport and exchange reactions, and 1 biomass reaction. Tables S1 and S2 in Supplementary Materials). 17.61% of the total open reading frames (ORFs), corresponding to 1282 genes of 7279 ORFs, were incorporated into the model. The SBML representation of the model can be downloaded from the supplemental files (File S1 in Supplementary Materials).

**Figure 2:**
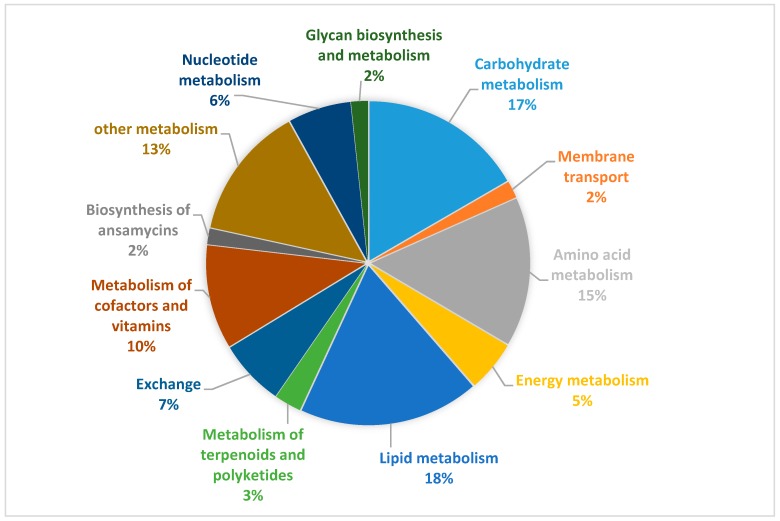
Percentage of reactions in each subsystem in *Aspm1282* model.

According to KEGG [36] and RAST [37] database, the 1399 reactions with functional annotation in *Aspm1282* are classified into eight subsystems, including carbohydrate metabolism, amino acid metabolism, energy metabolism, lipid metabolism, metabolism of terpenoids and polyketides, metabolism of cofactors and vitamins, biosynthesis of ansamycins, and other metabolism (Figure 2). 87% reactions have definite functional annotations, indicating a highly completed model among actinobacteria. The lipid metabolism is the largest subsystem with 18% reactions, followed by carbohydrate metabolism (17%), and amino acid metabolism (15%), totally accounting for half of the entire biological processes.

### 3.2. Aspm1282 Model Validation by the Prediction of Growth and Key Genes for Ansamitocin P-3 Biosynthesis

*Aspm1282* is so far the first reconstructed genome-scale model for *A. pretiosum*, there are no enough standard information can be used to validate the accuracy of the model, such as essential genes for *E. coli* and yeast model evaluation [38,39]. We compared simulated growth phenotypes of *Aspm1282* by FBA with the experimental data. The glucose uptake rate was set as 0.0912 mmol/gDW/h, which was the maximum measured uptake rate. We predicted the maximal biomass flux was 0.2019/h, similar with the experimental data, 0.1792/h, which showed that the model can simulate the growth of ATCC 31280 to some extent.

**Figure 3:**
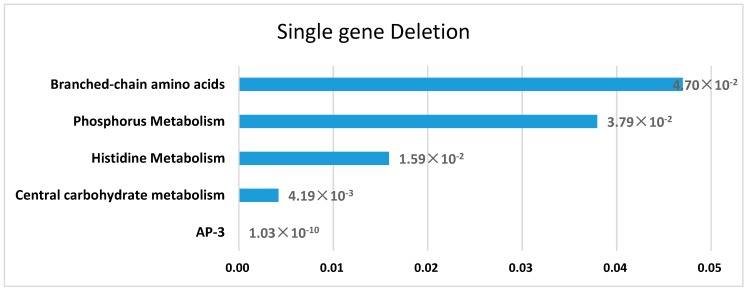
Enriched pathways of the genes essential for ansamitocin P-3 (AP-3) biosynthesis. The vertical axis represents enriched pathways, and the horizontal axis represents the *p*-value of each pathway.

To validate the effectiveness of *Aspm1282* in predicting AP-3 biosynthesis, essential gene analysis was performed by single-gene deletion, with the flux of AP-3 production as the objective function. There were 37 genes in the model predicted as influential genes, and 21 of them were found to be essential, whose deletions causing a decrease of AP-3 production more than 90% (Table S3 in Supplementary Materials). The enriched pathways [40] for influential genes is shown in Figure 3, and the genes in AP-3 biosynthesis pathway has the most significant effect on AP-3 production, which is consistent with previous finding [23]. In addition, central carbohydrate metabolism, histidine metabolism, and branched-chain amino acid pathway are also enriched with the influential genes, providing precursors for AP-3 biosynthesis, such as malonyl-CoA (coenzyme A), methylmalonyl-CoA, methoxymalonyl-ACP (acyl carrier protein), glutamate, and valine. Phosphorus metabolism involves the biosynthesis of ATP, which provides the necessary energy for the AP-3 product. The *Aspm1282* model efficiently reflects the metabolic characteristics of AP-3 biosynthesis.

To further validate the accuracy of the model, we compared the experimental data collected from published literatures (Table 3) with the phenotype predicted by *Aspm1282*. Previous experiments confirmed that the expression level of specific regulatory genes including *Asm8* and *Asm18*, and the concentrations of metabolites including Mg^2+^, glycerol, and ammonium have a dominant impact on the production of AP-3 [8,41,42,43,44,45,46]. The affected genes or enzymes by these regulatory genes and metabolites are summarized as in Table 2. Then, we carried out the robustness analysis for each key gene/enzyme and found that all genes/enzymes are positively correlated with the production of AP-3 (Text S1 in Supplementary Materials), which proved that the *Aspm1282* model can accurately reflect the effects of the key regulatory genes and metabolites on AP-3 biosynthesis.

### 3.3. Metabolic Shift of Ansamitocin P-3 High-Yield Mutant NXJ-24 During Fermentation Process by Condition-Specific Models

Since environment conditions have impact on the metabolic capability of strain [32], condition-specific models are required to reflect the particular metabolic phenotype by integration with omics data. NXJ-24 is a mutant of *A. pretiosum* ATCC 31280 with high production of AP-3 [23], and the transcriptome data of NXJ-24 mutant were obtained with samples collected at the first, second, third, and fifth days during fermentation process; to better understand the character of NXJ-24 in decline phase, we chose to sequence the transcriptome at the fifth day, instead of the fourth day. Here, we constructed condition-specific metabolic models during the fermentation process by integrating the transcriptome data using E-FLUX method to make comparisons of the specific metabolic capabilities and explore the potential factors that affect the production of AP-3. We generated the model for NXJ-24 mutant by knocking out of *asm30* in *Aspm1282* model, and the overexpression of *asm10* gene was automatically converted to higher flux bound by the mapping to transcriptome data with E-FLUX.

**Figure 4:**
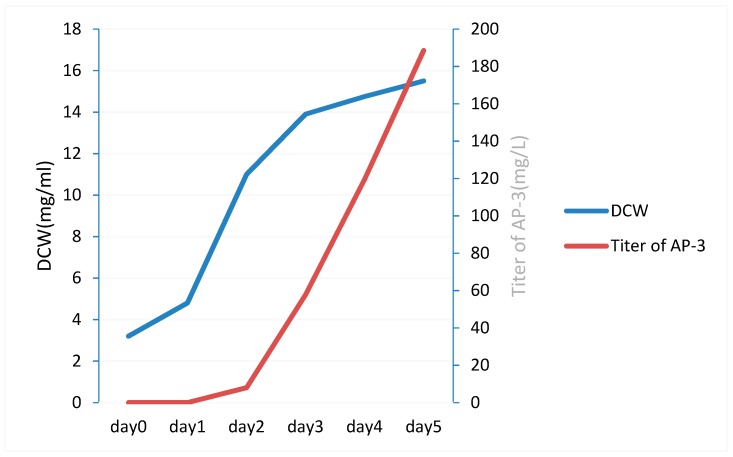
The dry cell weight (DCW) and titer of AP-3 in NXJ-24 mutant during fermentation process.

The growth rate, glucose uptake rate, and titer of AP-3 in each day of cultivation were experimentally measured (Figure 4). Glucose is considered to be the main carbon source for *A. pretiosum* [47], and the uptake rate of glucose has great impact on the energy and carbohydrate metabolism. The flux of glucose uptake in each day was set as a particular value, which was calculated from the measured residue glucose concentrations and DCW (dry cell weight) by experiment.

R_glucose_ = consumption_glucose_/(DCW·molecular weight_glucose_·time) (7)

Because simple FBA simulation with the biomass equation in the generic *Aspm1282* model as an objective function will result in no flux through AP-3 biosynthesis pathway, we added AP-3 in the biomass function to make a new objective in NXJ-24 specific models (Equation (8)). Then we can observe the transformation of flux distribution over time in ansamycin biosynthetic pathway in the high-yield mutant. The coefficients c of AP-3 component in the objective function were determined by the relative ratio between AP-3 titer and biomass measured by experiment in Figure 4.

Objective = Biomass + c × AP-3(8)

In the conditional specific models of day 1, day 2, day 3, and day 5, c is equal to 0, 0.0013, 0.0068, 0.0084 respectively.

**Figure 5:**
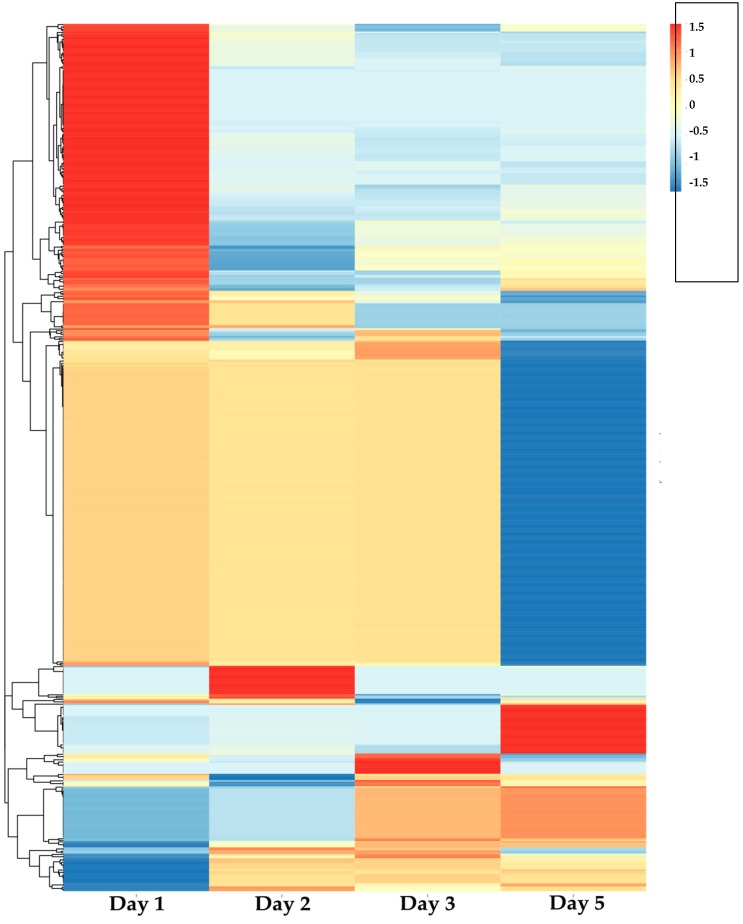
The flux distribution of NXJ-24 mutant during different fermentation process. The rows represent the reactions in the *Asmp1282*.

**Figure 6:**
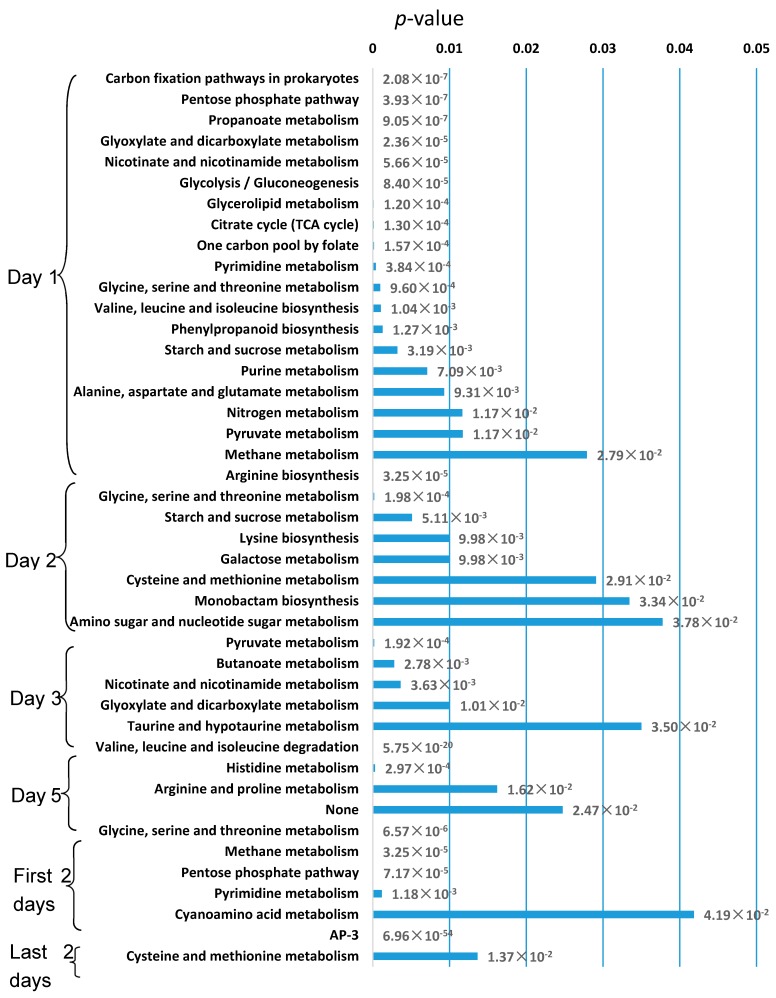
Enriched pathways of specifically active reactions in each day. The vertical axis represents enriched pathways, and the horizontal axis represents the *p*-value of enrich analysis of each pathway.

We used FBA to calculate the flux distribution of each specific model during cultivation process, as shown in Figure 5. We selected specific active reactions in each day, whose normalized flux was greater than 0.5 and lower than 0 in other days. Then we found the pathways enriched with active reactions, as shown in Figure 6. The number of active reactions and pathways on the first day is significantly more than other days, and most of them belong to the central carbon metabolism, nucleotide metabolism and energy metabolism, such as citrate cycle pathway, glycolysis/gluconeogenesis pathway, purine metabolism pathway, and carbon fixation pathways in prokaryotes. Since the central carbon metabolism, nucleotide metabolism, and energy metabolism are mainly related with growth and replication of cells, we call them growth related pathways thereafter. During the fermentation process, the percentage of growth related pathways in the enriched active pathways is decreased. For the first two days with higher growth rate, most of the active reactions belong to the growth related pathways. While for the last two days, very few growth related pathways were active, but the AP-3 biosynthesis pathway and cysteine and methionine metabolism were significantly active. This result showed that the replication, energy generation, and uptake of carbon source are the main objective in the first two days, and the capacity of growth is seriously declined in the last two days, while the AP-3 biosynthesis starts to increase.

We found the metabolic shift from growth associated pathways to other metabolism started from the second day of cultivation. Although the growth rate was highest on the second day, the active pathway started to transform from growth related pathways to other pathways, such as cysteine and methionine metabolism and secondary metabolism pathway. It indicated that the growth rate might reach maximum at the moment between the two measurements, but the metabolic mode has changed at the second measurement. We hope to get more precise metabolic shift pattern with highly frequent time-course measurements during cultivation in the future.

In addition, it has been reported that the production of AP-3 was improved in the pentose phosphate pathway weaken mutant [48]. Here we found pentose phosphate pathway was active in the first two days, but inactive in the last two days, when the AP-3 biosynthesis increased.

### 3.4. Relationship of Methionine Pathway and Ansamitocin P-3 Biosynthesis

The metabolic shift during fermentation process indicated that the biosynthesis of AP-3 is initiated when the cells sense stressful conditions, similar with most secondary metabolism. The significant high activity of cysteine and methionine metabolism in the last two days showed that this pathway plays an important role in the biosynthesis of AP-3. Methionine is the precursor of SAM (*S*-adenosyl-l-methionine), which is the key methyl donor for three steps of methylation reactions in AP-3 biosynthesis pathway. More significantly, one of the methylation reactions, catalyzed by *asm10*, was reported as the bottleneck in the production of AP-3, the increase in *asm10* can increase the AP-3 production by 93% [23,49].

**Figure 7:**
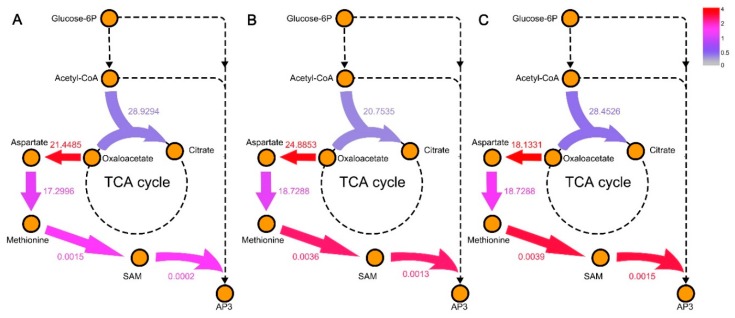
The flux change of methionine biosynthesis and tricarboxylic acid cycle (TCA cycle) in the specific models for day 2 (**A**), day 3 (**B**), and day 5 (**C**) compared with day 1. The values represent the flux through each reaction. The colored arrow represent the ratio of flux in each day to that of day 1. SAM: S-adenosyl-L-methionine.

To further explore the relationship among cysteine and methionine metabolism, cell growth, and AP-3 production, we simulated the biosynthetic velocity of key metabolites in methionine biosynthesis and tricarboxylic acid cycle (TCA cycle), as shown in Figure 7. The biosynthetic velocity of citrate in the first day was significantly higher than that in other days, while the velocity of aspartate, methionine, and SAM were lower. Most flux flow into the TCA cycle on the first day to meet the growth demand of the strain. Since the second day, the flux turned to flow into the biosynthesis of methionine pathway, which improve the concentration of SAM in the cell and contribute to the biosynthesis of AP-3 [50].

### 3.5. Strain Optimization for Ansamitocin P-3 Overproduction by Aspm1282 Model

Using the *Aspm1282* model, we aimed to identify potential engineering strategy for AP-3 overproduction. A series of computational strain optimization methods have been published, such as RobustKnock [51], OptGene [52], OptORF [53], GDLS [54], and FSEOF [55]. The meta-heuristic methods such as OptGene have been used widely, which aim to find the solution fulfilling the best optimized function, usually biomass-product coupled yield (BPCY). But sometimes BPCY remains 0, and the algorithm reports no feasible solution. To better characterize the coupling between biomass and target production, we proposed an improved objective function (Equation (9)) in our in silico strain design approach OptRAM, which can identify strategies for up-regulation, down-regulation or knockout of metabolic genes by simulated annealing algorithm.

(9) $$\mathrm{Obj}=\frac{\mathrm{Target}\times \mathrm{Growth}}{\mathrm{Substrate}}\times \left(1-\log\frac{\mathrm{Range}}{\mathrm{Target}}\right)$$

(10) $$\mathrm{Target}=\frac{V_{\max}+V_{\min}}{2},\;\mathrm{Range}=\frac{V_{\max}-V_{\min}}{2}.$$

Target means the average flux value of target product. Range is set to half of the interval between min and max target flux value.

**Figure 8:**
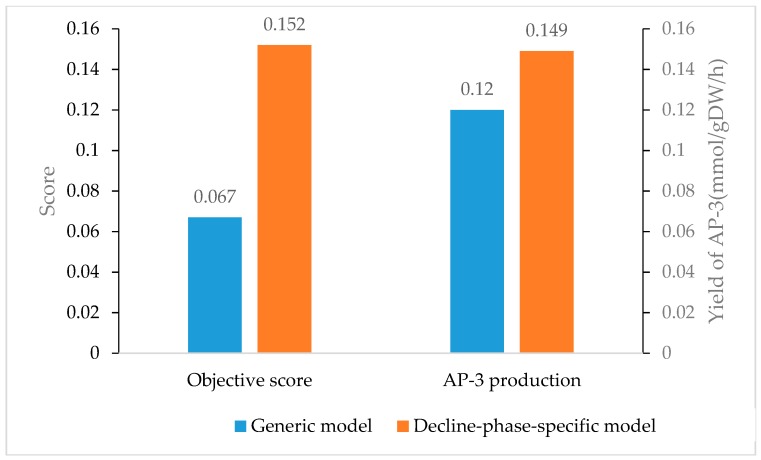
The mean objective score and mean AP-3 production predicted by generic *Aspm1282* model and decline-phase-specific model.

The other advantage of OptRAM is that we incorporated an evaluation mechanism to determine the best and feasible genetic modification solution, by considering growth rate, product of desired compound, and implementation cost. We ran OptRAM 25 times for the generic *Aspm1282* model and the decline-phase-specific model, respectively, integrating expression data of the wild-type strain on day 5 (Table S4 in Supplementary Materials). The average of objective score and AP-3 production flux of the two models were illustrated in Figure 8, which demonstrated that the decline-phase-specific model outperformed generic model with both higher AP-3 production and better coupling with growth. Therefore, the model integrated with gene expression data in decline phase could be more suitable to simulate the metabolic pattern of secondary-metabolite-biosynthesis strain.

**Figure 9:**
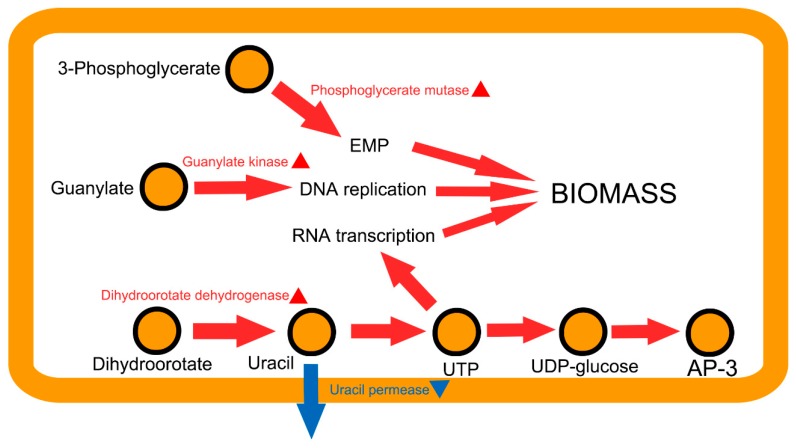
The mechanism of the optimized modification strategy to improve AP-3 and growth. The arrows represent flux rate of particular biological processes related with the biomass and AP-3 biosynthesis. The modification sites identified by OptRAM are labeled with triangle, and the red and blue ones mean overexpression or underexpression, respectively. Red and blue colored arrows represent up- or downregulation of the reactions in modified strain compared to the wild-type. AP-3: ansamitocin P-3; EMP: Embden Meyerhof Parnas pathway; UTP: uridine triphosphate; UDP: uridine diphosphate.

We selected the solution with the highest objective score among 25 simulations by decline-phase-specific model, which included four genetic modification sites. The detailed annotation and expression adjustment were shown in Table 4. The mechanism of the optimized modification strategy to improve AP-3 was illustrated in Figure 9. In this strategy, 42197.4.peg.5418 (guanylate kinase) and 42197.4.peg.5889 (uracil permease) are involved in nucleotide synthesis. The upregulation of 42197.4.peg.5418 can improve the biosynthesis of GTP (guanosine triphosphate) and thus promote the DNA replication. The down regulation of uracil permease can reduce the leakage of uracil to increase the flux rate of RNA synthesis to some extent. 42197.4.peg.3610 (phosphoglycerate mutase) catalyzes a critical step in glycolysis/gluconeogenesis pathway, whose upregulation can improve the growth rate of strain. These three genetic modification sites are all relevant to growth rate improvement. The other modification site, upregulation of 42197.4.peg.5886 (dihydroorotate dehydrogenase), can accelerate the biosynthesis of UDP (uridine diphosphate), which can not only enhance growth rate, but also improve the synthesis of UDP-glucose, a key precursor of AP-3. The combinatorial modification of the above four genes identified by computational simulation improved the theoretical flux of AP-3 synthesis from 0.201 to 0.372 mmol/gDW/h, and kept better growth meanwhile. It validated that the *Aspm1282* model is relatively accurate and useful for guiding strain design.

## 4. Discussion

### 4.1. The First Genome-Scale Metabolic Model of A. pretiosum

In this study, we reconstructed the first GSMM *Aspm1282* for *A. pretiosum* ATCC 31280, which provides a fundamental and useful system to study the metabolic mechanism and guide for AP-3 overproduction. The model *Aspm1282* covers 90.7% reactions with annotated genes, and 87% reactions with clear pathway information. Usually there are 50% genes with accurate functional annotation for actinobacteria, while our model accounts 87% metabolic genes with specific pathway function, which complements the interpretation of *A. pretiosum* genome and also provides a reference for other actinobacteria, such as *Streptomyces coelicolor* and *Sacchropolysora erythrae*. The effectiveness of this model has been confirmed by the positive effects of reported key factors and metabolites on AP-3 production. The lack of experimental information is still a limit for the validation of the model reconstructed here. Further experiments on *A. pretiosum* time-course growth and gene deletion should help us better refine and complete the model.

### 4.2. Metabolic Shift of Ansamitocin P-3 High-Yield Mutant NXJ-24 during Fermentation Process

As shown in Figure 5, it was obvious that the number of specific active reactions in the first day is significantly greater than the other days, which indicated that the strain is relative vigorous in the first day. Figure 6 illustrated that during the fermentation process, the percentage of the active growth related pathways decreased, and there is even no enriched growth related pathway in the last two days. The changes of flux distribution of condition-specific model can well reflect the metabolic shift from growth related pathway to other pathways such as amino acid metabolism and secondary metabolism pathway since the second day of cultivation. The AP-3 and cysteine and methionine metabolisms were enriched in the last two days, which indicated there are certain relationships among cell growth, activation of cysteine and methionine metabolism, and the biosynthesis of AP-3.

### 4.3. Up-Regulation of Methionine Biosynthetic Pathway May Be a Potential Strategy to Improve the Production of Ansamitocin P-3

Both the significantly active methionine pathway in the latter stage of fermentation and the relationship between methionine pathway and AP-3 biosynthesis indicated that the methionine pathway could be a potentially important pathway for AP-3 overproduction. It was reported that the *N*-methylation reaction, *S*-adenosyl-l-methionine + *N*-demethyl-ansamitocin P-3 → *S*-adenosyl-l-homocysteine + ansamitocin P-3 (named R09852 in KEGG database), catalyzed by Asm10, was one of the bottlenecks in AP-3 biosynthesis [23]. As a substrate of R09852, the biosynthesis velocity of SAM and its main precursor, methionine, could have a prominent impact on the production of AP-3. As shown in Figure 7A, it was obvious that with the change of metabolic pattern at the second day, the flux was diverted to aspartate from citrate. Because aspartate is the beginning of methionine pathway, this metabolic shift could promote the accumulation of methionine and thus increase the production of AP-3. For the predicted engineering strategy obtained from OptRAM, we also found gene 42197.4.peg.5105 (5-methyltetrahydrofolate-homocysteine methyltransferase) involved in methionine biosynthesis. As mentioned above, the up-regulation of it may improve the biosynthesis of SAM.

### 4.4. Application of the Reconstructed Model in Strain Design for Ansamitocin P-3 Overproduction

In actual industrial production, the target production efficiency is determined by both the biomass and flux of the target compound. Therefore, we set Obj as the evaluation of strain modification, which considers biomass and product simultaneously. We used OptRAM method to simulate *Aspm1282* model and identify a modification strategy for AP-3 overproduction, which enhances the EMP pathway (Embden Meyerhof Parnas pathway), reduces the release of uracil, and improves the biosynthesis of UTP and GTP. EMP is an important pathway that provides energy and carbon skeleton for biomass. The improvement of UTP and GTP biosynthesis can promote the replication of DNA and the transcription of RNA, both of which are essential for biomass. What is more, the biosynthesis of UTP directly affects the production of AP-3 through the biosynthesis of UDP-glucose, one of the precursors for AP-3. All these modification sites demonstrated that the strain optimization by our newly reconstructed model is of great potential for real application in AP-3 overproduction.

## Figures and Tables

**Table 1:** Perturbation code and the corresponding fold change (FC) of mutated gene expression.

Code		1	2	3	4	5
FC		2	4	8	16	32
Code	0	−1	−2	−3	−4	−5
FC	0.001	1/2	1/4	1/8	1/16	1/32

**Table 2:** Composition of Genome-Scale Metabolic Models (GSMM) *Aspm1282* for *Actinosynnema* *pretiosum* ATCC 31280.

Categories	Numbers
Genes	1282
Reactions	1669
Metabolites	1614
Open reading frames (ORFs)	7279
Exchange reactions	118
Transport reactions	30
Compartments	2
Subsystems	12

**Table 3:** The reported key regulators and metabolites for AP-3 production and the robustness simulation of corresponding enzymes in the model.

Regulator/Metabolite	Corresponding Enzymes in Model	Simulation Positively Correlated with AP-3
*Asm8*	*Asm23*, *Asm24*, *Asm43*, *Asm44*, *Asm45*	√
*Asm18*	*Asm21*, *AsmA*, *Asm43*	√
Mg^2+^	Methylmalonyl-CoA mutase, methylmalonyl-CoA carboxyltransferase	√
Glycerol	Phosphoglucomutase, *Asm14*, *Asm24*	√
Ammonium	*Asm14*, *Asm24*, *Asm43*, *Asm19*	√

**Table 4:** The predicted modification of *A. pretiosum* ATCC 31280 for AP-3 overproduction.

Genes	Enzymes	Modifications
42197.4.peg.3610	Phosphoglycerate mutase	Overexpression
42197.4.peg.5418	Guanylate kinase	Overexpression
42197.4.peg.5886	Dihydroorotate dehydrogenase	Overexpression
42197.4.peg.5889	Uracil permease	Underexpression

Objective Score = 0.388; Product = 0.372.

## Supplementary Materials

The following are available online at http://www.mdpi.com/2073-4425/9/7/364/s1, File S1: The SBML file of reconstructed genome-scale model *Aspm1282* for *A. pretiosum* ATCC 31280, Table S1: List of reactions in *Aspm1282* model, Table S2: Compositions of biomass for *A. pretiosum* ATCC 31280, Table S3: The effects of single gene deletions on AP-3 biosynthesis flux predicted by *Aspm1282* model, Table S4: The strain optimization solutions of OptRAM for improving AP-3 by generic model and Decline-phase-specific model, Text S1: The positive correlation between AP-3 production with enzyme-catalyzing-reactions affected by literature-reported regulatory genes and metabolites for AP-3.
