# Supplementary material for: Genome-Scale Metabolic Model of Actinosynnema pretiosum ATCC 31280 and Its Application for Ansamitocin P-3 Production Improvement
Source: Genes (Basel). 2018 Jul 20;9(7):364. doi: 10.3390/genes9070364 (PMC6070911; doi:10.3390/genes9070364)
Supplement: Supplementary file 1 [file genes-09-00364-s001.zip › supplymentary file/Text S1.doc]

**Text S1.** The effects of single gene deletions on AP-3 biosynthesis flux predicted by *Aspm1282* model

The result of robustness analysis can prove the correlation of most genes in Table 3.

**Figure S1.** The robustness analysis result between yield of AP-3 and reactions catalyzed by AsmA,Asm14,Asm19,Asm21,Asm23,Asm24,Asm43,Asm44,Asm45

**Figure S2.** The robustness analysis result between yield of AP-3 and rxn00678_c0 catalyzed by methylmalonyl-CoA carboxyltransferase

**Figure S3.** The robustness analysis result between yield of AP-3 and rxn00602_c0 catalyzed by Methylmalonyl-CoA mutase

Because of catalyzing many [isoenzyme](javascript:void(0);)s, the robustness analysis cannot prove the positive correlation between yield of AP-3 and phosphoglucomutase. However, the result of single gene deletion can prove the essential role of phosphoglucomutase for AP-3.

**Table S5.** Part of Table S3, the result of single gene deletion of phosphoglucomutase.

| **Gene** | **AP-3 product** | **Enzyme** |
| --- | --- | --- |
| 42197.4.peg.1859 | 0 | Phosphoglucomutase |
